# 1,3-Propanediol dehydrogenases in *Lactobacillus reuteri*: impact on central metabolism and 3-hydroxypropionaldehyde production

**DOI:** 10.1186/1475-2859-10-61

**Published:** 2011-08-03

**Authors:** Marc J A Stevens, Sabine Vollenweider, Leo Meile, Christophe Lacroix

**Affiliations:** 1 Laboratory of Food Biotechnology, Institute of Food, Nutrition and Health, ETH Zurich, Schmelzbergstrasse 7, 8092 Zurich, Switzerland; 2 Flavour Science & Technology, Natural Flavour Ingredients, Givaudan Schweiz AG, Ueberlandstrasse 138, CH-8600 Dübendorf, Switzerland

## Abstract

**Background:**

*Lactobacillus reuteri *metabolizes glycerol to 3-hydroxypropionaldehyde (3-HPA) and further to 1,3-propanediol (1,3-PDO), the latter step catalysed by a propanediol dehydrogenase (PDH). The last step in this pathway regenerates NAD^+ ^and enables therefore the energetically more favourable production of acetate over ethanol during growth on glucose.

**Results:**

A search throughout the genome of *L. reuteri *DSM 20016 revealed two putative PDHs encoded by ORFs lr_0030 and lr_1734. ORF lr_1734 is situated in the *pdu *operon encoding the glycerol conversion machinery and therefore likely involved in 1,3-PDO formation. ORF lr_0030 has not been associated with PDH-activity so far. To elucidate the role of these two PDHs, gene deletion mutant strains were constructed. Growth behaviour on glucose was comparable between the wild type and both mutant strains. However, on glucose + glycerol, the exponential growth rate of Δlr_0030 was lower compared to the wild type and the lr_1734 mutant. Furthermore, glycerol addition resulted in decreased ethanol production in the wild type and Δlr_1734, but not in Δlr_0030. PDH activity measurements using 3-HPA as a substrate revealed lower activity of Δlr_0030 extracts from exponential growing cells compared to wild type and Δlr_1734 extracts.

During biotechnological 3-HPA production using non-growing cells, the ratio 3-HPA to 1,3-PDO was approximately 7 in the wild type and Δlr_0030, whereas this ratio was 12.5 in the mutant Δlr_1734.

**Conclusion:**

The enzyme encoded by lr_0030 plays a pivotal role in 3-HPA conversion in exponential growing *L. reuteri *cells. The enzyme encoded by lr_1734 is active during 3-HPA production by non-growing cells and this enzyme is a useful target to enhance 3-HPA production and minimize formation of the by-product 1,3-PDO.

## Introduction

*Lactobacillus reuteri *is a heterofermentative lactic acid bacterium (LAB) encountered in a variety of fermented foods like sourdough, meat, and dairy products [1-3]. Furthermore it is a natural inhabitant of the gastro-intestinal (GI) and urogenital tract of humans and other animals [4-8]. Some strains of *L. reuteri *strains exhibit probiotic properties and were developed as probiotic products [9].

Most *L. reuteri *strains produce and excrete reuterin, an antimicrobial compound consisting of hydrated, non-hydrated, and dimeric forms of 3-hydroxypropionaldehyde or 3-HPA [10,11]. Remarkably, the capability to produce reuterin seems to be absent in most *L. reuteri *rodent isolates [12]. Reuterin is active against a large range of micro-organisms and is assumed to give *L. reuteri *a competitive advantage in its ecological niches as e.g. the mammalian GI-tract. Furthermore, 3-HPA produced by the food grade LAB *L. reuteri *is of industrial interest as it has potential use as food preservative, sanitizing agent, and as precursor for the production of chemicals like acrylic acid and polymers [13,14]. The reuterin form 3-HPA is produced from glycerol in a vitamin B_12_-mediated reaction catalysed by a glycerol dehydratase [15]. Synthesis of 3-HPA is a complex multi-protein biological process and takes place in micro-compartments that presumably form a barrier to protect the cytosol against 3-HPA [16,17]. Biotechnological production of 3-HPA is best-done in a two-stage process, in which biomass is produced in the first stage and glycerol is converted to 3-HPA in the second stage using concentrated cells from stage one. This method results in rapid glycerol conversion and 3-HPA concentrations of up to 235 mM out of 400 mM glycerol [18]. However, apart from 3-HPA, the by-product 1,3-propanediol (1,3-PDO) accumulates in the medium, resulting in lower yields and more complicated purification of 3-HPA.

**Figure 1:**
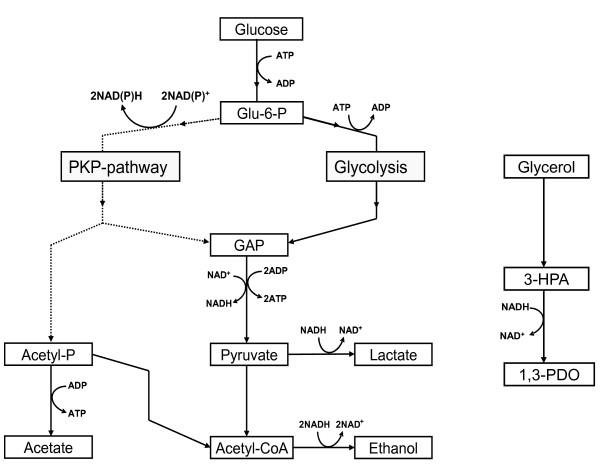
**Glucose and glycerol utilisation by *L. reuteri***. The glycolysis is indicated with black lines, the phosphoketolase (PKP)-pathway with dashed lines. The reduced equivalents produced in the phosphoketolase pathway can only be balanced via ethanol production or via an external electron acceptor as e.g. 3-HPA.

*L. reuteri *uses both the Embden-Meyerhof pathway (EMP) and phosphoketolase pathway (PKP) when growing on glucose and produces lactate and ethanol as major fermentation end-products (Figure 1; [19]). However, growth on glucose is limited due to a redox imbalance, which results in ethanol formation over the energetically more favourable acetate formation. Addition of an electron acceptor such as fructose restores the redox balance resulting in acetate formation, 1 ATP gain, and higher growth rate and biomass production [19]. Another suitable electron acceptor is glycerol-derived 3-HPA. 3-HPA can be converted to 1,3-PDO in a reaction catalysed by 1,3-propanediol dehydrogenases (PDH) thereby regenerating one NAD^+ ^molecule (Figure 1). Supplementation of the growth medium with glycerol indeed results in a higher growth rate and biomass production on glucose [20].

In this work two genes encoding putative 1,3-propanediol dehydrogenases (PDHs) were identified in the genome of *L. reuteri *DSM 20016. Mutagenesis of the genes and subsequent physiological analyses revealed that one PDH is mainly active during exponential growth, whereas the other PDH is involved in 3-HPA conversion in non-growing cells.

## Material and Methods

### Bacterial strains, media, and growth conditions

Bacterial strains and plasmids used in this study are listed in table 1. *E. coli *DH5α was used as an intermediate cloning host and grown aerobically at 37°C in TY-medium [21]. When appropriate, chloramphenicol was added to a final concentration of 8 μg/ml. *L. reuteri *strains and their derivatives were grown anaerobically in sterile filtered 0.5x or 1x MRS broth [22] or on autoclaved MRS-agar plates at 37° C. TY-medium was obtained from Difco Laboratories (Detroit, USA), MRS medium was obtained from Biolife Italiana (Milan, Italy). Anaerobic conditions in jars were maintained using the AnaeroGen oxygen scavenger system (Oxoid, Basingstoke, United Kingdom). Growth was monitored by measuring the optical density at 600 nm (OD_600_) using a spectrophotometer UVIKON 810 P (Tegimenta AG, Rotkreuz, Switzerland).

**Table 1:** Strains and plasmids used in this study and their relevant characteristics

*Material*	***Relevant features***^a^	*Source*
**Strains**		
*E. coli*		
DH5α	Cloning host	[37]
*L. reuteri*		
DSM 20018	Wild type strain, human intestinal isolate	DSMZ^b^
SD2112	Wild type strain, human milk isolate	ATCC^c^
LFB1001	lr_1734 gene replacement (lr_1734::P_32_*cat*) derivative of *L. reuteri *DSM20016, Cm^R^.	This work
LFB1002	lr_0030 gene replacement (lr_0030::P_32_*cat*) derivative of *L. reuteri *DSM20016, Cm^R^.	This work
LFB1003	lr_1734 gene replacement (lr_1734::P_32_*cat*) derivative of *L. reuteri *SD2112, Cm^R^.	This work
**Plasmids**		
pNZ5319	Cm^R^, Em^R^, gene replacement mutagenesis vector.	[25]
pLFB1001	Cm^R^, Em^R^, pNZ5319 derivative containing 1.1 kb 3' flanking region of lr_1734.	This work
pLFB1002	Cm^R^, Em^R^, pNZ5319 derivative lr_1734::*cat *gene replacement mutagenesis vector containing 5'- and 3'-flanking regions of lr_1734.	This work
pLFB1003	Cm^R^, Em^R^, pNZ5319 derivative containing 1.2 kb 5' flanking region of lr_0030.	This work
pLFB1004	Cm^R^, Em^R^, pNZ5319 derivative lr_0030*::*cat gene replacement mutagenesis vector containing 5'- and 3'-flanking regions of lr_0030.	This work

^a^Cm^R^, chloramphenicol resistant; Em^R^, erythromycin resistant.

^b^DSMZ: German Collection of Microorganisms and Cell Cultures.

^c^ATCC: American Type Culture Collection.

### DNA manipulations and gene disruption

Molecular cloning and DNA manipulations were essentially performed as described by Sambrook et al. [21]. Large scale plasmid DNA isolations from *E. coli *were performed using a Maxiprep Kit (Qiagen, Basel, Switzerland). Chromosomal DNA isolation from *L. reuteri *was performed using a phenol-chloroform extraction method based on a protocol for *Lactobacillus plantarum *[23]. Restriction enzymes and Phusion-polymerase were obtained from New England Biolabs (Frankfurt am Main, Germany) and T4-ligase from Invitrogen (Basel, Switzerland). Primers were purchased from Microsynth (Balgach, Switzerland).

### Construction of plasmids and strains

To delete the *pduQ *gene encoded by ORF lr_1734 in *L. reuteri *DSM 20016, a double-cross-over (DCO) gene replacement strategy was used which results in replacement of the target gene by the chloramphenicol resistance gene-cassette P_32_*cat *[24] in the same orientation as the target gene (Additional file 1, Figure S1). A 1.1-kb fragment of the downstream region of lr_1734 was amplified using a proof-reading DNA-polymerase (Phusion) and the primers *1734-D-5' *and *1734-D-3' *(Table 2). The downstream fragment was cloned into the Ecl136II site of the lactobacilli gene replacement vector pNZ5319 [25], resulting in vector pLFB1001. Subsequently, a 1.1-kb fragment of the upstream region of lr_1734 was amplified using primers *1734-U-5' *and *1734-U-3' *(Table 2) and the fragment was cloned into the SwaI-site of pLFB1001, resulting in the lr_1734 gene replacement vector pLFB1002, containing 1.1-kb up- and downstream regions of lr_1734. Correct plasmid construction was checked by PCR and restriction analyses.

**Table 2:** Primers used in this study

**Name**:	**Sequence**:	**Name**:	**Sequence**:
*1734-U-3'*	5'-CCGGGTTGGCATACTATATTTT-3'	*1734-U-5'*	5'-GATTCTTGAACCAGTTGTAGAAAAC-3'
*1734-D-5'*	5'-GAAAGATGCAACATTCCCTGG-3'	*Con-cat-for*	5'-GATAGGCCTAATGACTGGCT-3'
*1734-D-3'*	5'-TCACCAATTCCGGCTGTAAA-3'	*Con-cat-rev*	5'-CTCTTCCAATTGTCTAAATC-3'
*0030-U-5'*	5'-TATTTATCGTTGTTAGCGATGG-3'	*Con-1734-5'*	5'-AATGCCTTGTACAACACTCC-3'
*0030-U-3'*	5'-CCAGGACCAAAGAATTCACAC-3'	*Con-1734-3'*	5'-TCGTCAATTTTGACTCCAAG-3'
*0030-D-5'*	5'-AGTCGGATCCGCACGGACTAAGATGGTTGA-3'	*Con-0030-5'*	5'-CGTTTGGACGTTTTACCTTACC-3'
*0030-D-3'*	5'-AGTCGAGCTCACGCTGTCAACCCAATACTT-3'	*Con-0030-3'*	5'-CTGATTTTGCGCGTTTCCTCTCTC-3'

A similar strategy was used for the deletion of ORF lr_0030. The primers *0030-U-5' *and *0030-U-3' *(Table 2) were used to amplify a 1.2-kb upstream fragment of lr_0030 and this fragment was cloned into the SwaI site of pNZ5319, resulting in pLFB1003. Similarly, a 1.1-kb downstream fragment of lr_0030 was amplified using the primer set *0030-D-5' *and *0030-D-3' *(Table 2) and this fragment was cloned into the Ecl136II-site of pLFB1003, resulting in the gene replacement vector pLFB1004 for ORF lr_0030. The restriction sites introduced in the primers *0030-D-5' *and *0030-D-3' *were not used for cloning.

The gene deletion vectors were transformed to *L. reuteri *as described previously [26] and plasmid-integrants were selected anaerobically at 37°C on MRS plates complemented with 8 μg/ml chloramphenicol. The anticipated genetic organization after correct gene replacement results in a chloramphenicol resistant but erythromycin sensitive phenotype. Colonies were therefore picked and transferred to MRS plates containing 10 μg/ml erythromycin and grown anaerobically at 37°C overnight. Integrants displaying a chloramphenicol resistant an erythromycin sensitive phenotype were checked by PCR using universal-primers annealing in the *cat*-gene (primers *Con-cat-for *and *Con-cat-rev*, table 2) and a site-specific primer annealing outside of the chromosomal region used for homologous recombination (*con-*primers, Table 2). Integrants showing the correct phenotype and positive PCR analyses were streaked on MRS with chloramphenicol and a single colony isolate was checked again by PCR (Additional file 1, Figure S1).

### Production and purification of 3-hydroxypropionaldehyde

All *L. reuteri *strains were grown in MRS supplemented with 35 mM glycerol (Sigma-Aldrich, Buchs, Switzerland) at 37°C to an OD_600 _of approximately 8.0, representing the early stationary phase. To obtain cells with comparable metabolic activity, 10 mM glucose and 20 mM glycerol (end-concentrations) were added to the culture and cells were reactivated for 30 minutes at 37°C. Subsequently, cells were harvested (4,000g, 10', RT) and washed once in 10 mM KPO_4_-buffer, pH 7.0. Productions were performed in 250 mM aqueous glycerol solution at an OD_600 _of approximately 60 for one hour. Samples for HPLC were centrifuged (12,000g, 5', 4°C), sterile filtered, and stored at 4°C until further analyses.

To purify 3-HPA, production supernatants were lyophilized and 3-HPA was subsequently purified by chromatography as described previously [27].

### Enzymatic analysis

Cell free supernatants were prepared as follows: cells (50 ml) were harvested from an exponentially growing culture at OD_600 _= 1.0, resuspended in 1/100 volume MRS broth and transferred to a screw-cap tube containing 500 mg zirconium beads. Cells were disrupted by 3 times 30" bead-beat treatments in a fast prep device (MP Biomedicals, Illkirch, France) interspaced by cooling on ice. Cell debris and beads were removed by centrifugation (12,000g, 5', 4°C), and the cell free supernatant containing the cytosolic proteins was transferred to a new tube and stored at 4°C until further use.

Conversion rate of 3-HPA to 1,3-PDO was determined by measuring the decrease of NADH_2_-absorption at 340 nm in a spectrophotometer UVIKON 810P. The reaction mixture contained 12.5 mM 3-HPA and 480 μM NADH_2 _(Sigma-Aldrich) in 50 mM Tris-HCl buffer pH 7.2. The reaction was initiated by addition of 20 μl cell free supernatant (approx. 40 μg protein) and specific activity was defined as the decrease of NADH_2 _in μmol mg_prot_^-1 ^min^-1^.

Protein concentrations were determined as described by Bradford [28].

### Determination of metabolic compounds

Concentrations of 3-HPA, glycerol, and 1,3-PDO were determined by HPLC on an Aminex HPX-87H column as described previously [29]. Concentrations of sugars and end-fermentation products were determined on the same column in a separate run with identical settings.

## Results

### Identification of putative 1,3-PDO dehydrogenases in *L. reuteri *DSM 20016

The complete genome sequence of *L. reuteri *strain DSM 20016 has been determined [30] and therefore we used this strain in the current study. To identify genes involved in 3-HPA to 1,3-PDO conversion, we searched for putative 1,3-propanediol dehydrogenases in the genome of *L. reuteri *DSM 20016. A BLASTP analysis using an experimentally verified PDH (PduQ) from *Salmonella typhimurium *as a query [31], revealed the presence of 3 putative PDHs (cut-off E value < 1exp-20). The best hit was a protein encoded by ORF lr_1734 (Table 3). Interestingly this gene is situated in the *pdu *operon encoding the genes for 3-HPA production [16], suggesting indeed involvement of the corresponding enzyme in 3-HPA conversion. The second hit, encoded by lr_0321, is twice the size of PduQ from *S. typhimurium *and is annotated as the enzyme involved in the conversion of acetyl-CoA to acetaldehyde [32]. The third hit, encoded by ORF lr_0030, is a protein comparable in size with PduQ from *Salmonella *and with the gene product of lr_1734 (Table 3). Homologous of this ORF are found in a number of lactobacilli, both reuterin and no-reuterin producers, although the genetic organisation is not conserved (data not shown).

**Table 3:** Results of BLASTP-search for putative 1,3-propenediol dehydrogenases (PDHs) in *L. reuteri *DSM 20016

*ORF*	*Size*	*Significance (E)*	*Identities*	*Positives*	*Gaps*	^*a*^*Exponential (2*^*n*^*)*	^*a*^*Stationary (2*^*n*^*)*
*lr_0030*	390 AA	7exp-42	117 of 348 (33%)	176 of 348 (50%)	15	1.43	1.52
*lr_0321*	878 AA	6exp-57	133 of 348 (38%)	193 of 348 (55%)	37	-2.01	-6.18
*lr_1734*	373 AA	2exp-71	129 of 275 (46%)	181 of 275 (65%)	1	0.90	0.62

^a^*Relative expression of the genes grown in the presence of glycerol compared to growth without glycerol in the exponential and stationary growth phase (data taken from *[33]).

A genome-wide transcription analysis of *L. reuteri *cells grown on glucose in the presence of glycerol compared to cells grown without glycerol revealed an about 3 times higher expression of ORF lr_1734 and lr_0030 in the presence of glycerol in both exponential and stationary cells (Table 3, [33]). In contrast, lr_0321 was down regulated by a factor >4 in the presence glycerol (Table 3), indicating that the corresponding enzyme is not involved in conversion of 3-HPA to 1,3-PDO. Therefore the combined BLAST and transcriptional analyses strongly suggest that *L. reuteri *DSM 20016 possesses two proteins involved in the conversion of 3-HPA to 1,3-PDO encoded by lr_1734 and lr_0030.

### Growth characteristics of two propanediol dehydrogenase mutants

To elucidate the 1,3-propanediol dehydrogenases activity of lr_0030 and lr_1734, two deletion strains were constructed using a double cross-over gene-replacement strategy resulting in strain LFB1001 (lr_1734::P_32_*cat*) and LFB1002 (lr_0030:: P_32_*cat *; Table 1). Glycerol conversion to 3-HPA and further to 1,3-PDO regenerates NAD^+ ^and has therefore directly an impact on the central pyruvate metabolism and ATP production in glucose growing *L. reuteri*. Addition of glycerol therefore results in faster growth and higher biomass production [20,33]. We grew the wild type DSM 20016 and the two mutant derivatives anaerobically in 0.5 × MRS medium with or without glycerol. Glycerol was added to a concentration of 35 mM and 0.5 × MRS was chosen because the differences between strains were more pronounced compared to standard MRS. The exponential growth rates without glycerol were similar for the wild type (μ = 0.73 h^-1^) and its mutant derivatives LFB1001 (μ = 0.75 h^-1^) and LFB1002 (μ = 0.79 h^-1^; Table 4). Addition of glycerol led to an increased growth rate in the wild type (μ = 0.87 h^-1^, *p *= 0.02), but not in strain LFB1001 (μ = 0.84 h^-1^, *p *= 0.13) and LFB1002 (μ = 0.78 h^-1^, *p *= 0.81), suggesting that these strain are less effective in regeneration of NAD^+ ^by using 3-HPA as acceptor.

**Table 4:** Maximum growth rate of *L. reuteri *DSM 20016 and its mutant derivatives

*Strain*	***No glycerol [h***^***-1***^***]***	***With glycerol [h***^***-1***^***]***	*Significance of increase in growth rate*
DSM20016 *wild type*	0.73 ± 0.01	0.87 ± 0.03	*p *= 0.02
LFB1001 (*Δlr_1734*)	0.75 ± 0.05	0.84 ± 0.00	*p *= 0.13
LFB1002 (*Δlr_0030*)	0.79 ± 0.05	0.78 ± 0.03	*p *= 0.81

The higher growth rates on glycerol supplemented medium suggest different ATP production rates and as a consequence different fermentation end-product yields i.e. acetate over ethanol production. Therefore the concentration of metabolites was determined in the culture supernatants at the early stationary growth phase. However, determination of *de novo *acetate production is hampered by the high amounts of acetate already present in the fermentation medium and because acetate and 3-HPA have virtually the same retention time on the HPLC column. When grown in absence of glycerol, glucose consumption and lactate and ethanol production was similar for each of the three strains (Table 5). In the presence of glycerol, no difference was observed in glucose consumption and lactate production between the strains, whereas glycerol was completely consumed by all the 3 strains (Table 5). However, a clear difference was observed in ethanol production. The wild type and LFB1001 (Δlr_1734) produced 20 mM ethanol (Table 5), whereas ethanol production in LFB1002 (Δlr_0030) was 32 mM, significantly higher compared to the wild type (*p *= 0.01) and LFB1001 (*p *= 0.04). This suggests that the wild type and LFB1001 need less ethanol production for the regeneration of NAD^+ ^compared to LFB1002.

**Table 5:** Glucose and glycerol consumption and end-product formation in *L. reuteri *DSM 20016 and its mutant derivatives LFB1001 and LFB1002 grown with and without glycerol^a^

*Strain*	*Glucose (Consumption)*	*Glycerol (Consumption)*	*lactate*	*ethanol*	*1,3-PDO*
*without glycerol*					
DSM 20016 (*wild type)*	- 31.8 ± 3.6	n.a	36.6 ± 1.8	62.7 ± 7.5	n.d.
LFB1001 (*Δlr_1734*)	- 32.1 ± 3.6	n.a.	36.8 ± 3.0	62.8 ± 7.6	n.d.
LFB1002 (*Δlr_0030*)	- 34.2 ± 0.4	n.a.	38.6 ± 0.8	60.9 ± 5.8	n.d.
*with 35 mM glycerol*					
DSM 20016 (*wild type)*	- 24.8 ± 1.8	- 34.5 ± 3.8	27.1 ± 2.5	20.5 ± 5.1	19.8 ± 0.8
LFB1001 (*Δlr_1734*)	- 24.8 ± 1.6	- 34.5 ± 4.8	26.4 ± 2.3	20.5 ± 3.7	19.1 ± 0.2
LFB1002 (*Δlr_0030*)	- 24.9 ± 2.6	- 34.5 ± 4.0	27.1 ± 2.8	32.2 ± 4.9^b^	16.3 ± 0.5^b^

^a^*Average values and standard deviations from duplicate experiments are given in mM. n.a.: not applicable, n.d.: not detected.*

^b^*Letters above values indicate significant difference (p < 0.05) compared to the two other strains in a t-test.*

If the wild type and LFB1001 produce less ethanol, they must regenerate NAD^+ ^via another pathway, probably via conversion of 3-HPA to 1,3-PDO. Indeed 1,3-PDO is produced by both strains up to 20 mM (Table 5). Production of 1,3-PDO in LFB1002 was lower (16 mM), which correlates with the observed higher ethanol production by this strain.

### Conversion of 3-HPA to 1,3 PDO in the mutant strains

**Figure 2:**
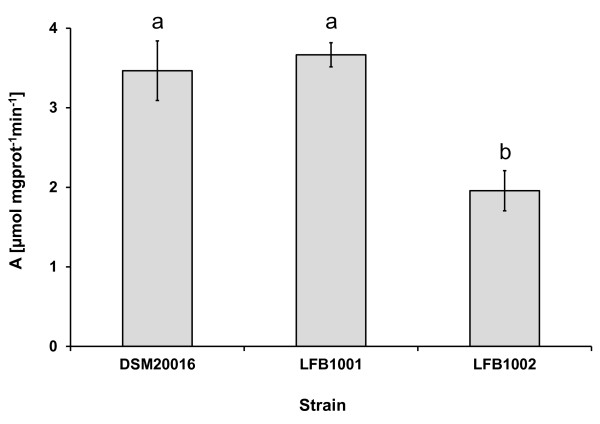
**Conversion rate of 3-HPA to 1,3-PDO by *L. reuteri *strains**. The conversion of 3-HPA to 1,3-PDO as determined by NADH_2 _oxidation activity of cell free extracts of DSM 20016 (wild type), LFB1001 (Δlr_1734) and LFB1002 (Δlr_0030). Averages of duplicate experiments are shown. Different letters above columns indicate significant difference (*p *< 0.05) in a t-test.

To elucidate if the altered end-fermentation pattern of LFB1002 on glycerol is due to a limitation in the conversion of 3-HPA to 1,3-PDO, the capability of 3-HPA conversion of the exponential growing wild type and the two mutants was tested. When grown in the presence of glycerol, the specific conversion rate of 3-HPA to 1,3-PDO was similar in the wild type and strain LFB1001 at a rate of approximately 3.5 μmol mg_prot_^-1 ^min^-1 ^(Figure 2). Strain LFB1002 displayed a lower rate of 1.96 μmol mg_prot_^-1 ^min^-1 ^(Figure 2), a significant lower conversion compared to the wild type (*p *= 0.042) and strain LFB1001 (*p *= 0.025). Cells grown without glycerol displayed no or minimal conversion of 3-HPA to 1,3-PDO with a specific conversion rate of 0.02-0.04 μmol mg_prot_^-1 ^min^-1 ^(data not shown), approximately 100 times lower compared to cells grown in the presence of glycerol. Furthermore, no significant differences in conversion rates could be detected between the wild type and both mutant strains.

The lower enzymatic activity of LFB1002 extracts from exponential growing cells shows that ORF lr_0030 encodes a gene product involved in 3-HPA conversion in cells exponentially growing in the presence of glycerol.

### Role of the PDHs during 3-HPA production

Biotechnological production of 3-HPA is possible using a two-stage process in which biomass production takes place in a first stage and bioconversion of glycerol in a second stage using concentrated cells from stage one. The wild type strain DSM 20016 and its two mutant derivatives were grown until the early stationary phase and subsequently productions were performed. The wild type produced 180 mM 3-HPA from 250 mM glycerol after one hour (Table 6). Strain LFB1001 (Δlr_1734) produced 200 mM whereas and LFB1002 (Δlr_0030) produced only 126 mM 3-HPA (Table 6). The formation of the by-product 1,3-PDO was 24.7 mM by the wild type (Table 6). Strain LFB1001 produced 16.0 mM 1,3-PDO (Table 6), resulting in a significant higher ratio of 3-HPA to 1,3-PDO compared to the wild type (*p *= 0.03), showing that lr_1734 encodes a PDH mainly active in non-growing cells. Strain LFB1002 produced similar amounts of 1,3-PDO as strain LFB1001, 16.7 mM, but the relative production of 3-HPA to 1,3-PDO was similar to that in the wild type (Table 6).

**Table 6:** 3-HPA and 1,3-PDO production out of 250 mM glycerol by *L. reuteri *wild type and mutant strains^a^

*Strain*	*3-HPA (mM)*	*1,3-PDO(mM)*	*Ratio 3-HPA/1,3-PDO*
DSM 20016 (*wild type)*	180.3 ± 11.9	24.7 ± 6.8	7.3
LFB1001 (*Δlr_1734*)	200.7 ± 8.2	16.0 ± 1.3	12.5^b^
LFB1002 (*Δlr_0030*)	125.7 ± 8.8	16.0 ± 1.0	7.9
SD2112 (*wild type)*	177.6 ± 15.1	15.0 ± 0.3	11.8
LFB1003 (*Δlr_1734*)	236.4 ± 2.0	6.8 ± 0.3	34.7^b^

^a^*Mean values from duplicate experiments plus standard deviation are presented.*

^b^*Letters above values indicate significant difference (p <0.05) in a t-test compared to the parental strain.*

*L. reuteri *SD2112 (ATCC 53608) is commonly used for 3-HPA production [18,34]. Therefore we constructed a knock-out strain in the lr_1734 homologous of strain SD2112, using the same gene replacement vector as for strain LFB1001. The resulting mutant, designated LFB1003 (Table 1), was applied in 3-HPA-production and the 1,3-PDO and 3-HPA profiles were compared to those in its parental strain SD2112 (Table 6). In parallel to the 3-HPA production profile of DSM 20016 compared to its Δlr_1734 mutant derivative LFB1001, strain LFB1003 produced smaller amounts of 1,3-PDO compared to its parental wild type, an effect even more pronounced compared to DSM 20016 (Table 6). The mutagenesis results in a significant increased 3-HPA/1,3-PDO ratio compared to the wild type strain (*p *= 0.02). This decrease of 1,3-PDO production in LFB1003 confirms that ORF lr_1734 in DSM 20016 encodes an enzyme involved in 1,3-PDO formation during 3-HPA production by resting cells.

## Discussion

*L. reuteri *has the unique capability among LAB to produce and excrete large amounts of 3-HPA, an intermediate of the glycerol reductive pathway. Engineering of metabolic pathways via road-blocking is a suitable method for minimizing by-product formation. However, deletion of a gene encoding the undesired enzymatic activity is not always successful as other enzymes might fulfil the deleted function. Indeed we identified more than one gene encoding for conversion of 3-HPA to 1,3-PDO, but deletion of only one of these two ORFs (lr_1734) resulted in decreased formation of the undesired product 1,3-PDO during 3-HPA production.

Glycerol derived 3-HPA can be used as an electron acceptor by *L. reuteri*, allowing the bacterium to close its NAD^+^/NADH_2_-balance and to produce acetate plus one additional ATP (Figure 1). Supplementation of the growth medium with glycerol leads to a higher growth on glucose by *L. reuteri *[33], as also observed in this study. Strain LFB1002 (Δlr_0030) did not display an increased growth rate after addition of glycerol, suggesting an impaired NAD^+ ^regeneration via 3-HPA conversion. *L. reuteri *DSM 20016 can grow up to 3-HPA concentration of 50 mM [26]. The low initial glycerol concentration (35 mM) does not result in such 3-HPA concentrations in the medium, making growth inhibition by accumulation of 3-HPA unlikely. Furthermore, addition of an electron acceptor leads to a shift in the metabolite production from ethanol to acetate [19,20]. Such a shift was clearly observed in the wild type and LFB1001 but not in LFB1002. Glucose consumption in the presence of glycerol was similar in all three strains (Table 5) and hence the different growth behaviour and fermentation pattern of LFB1002 indicates impaired 3-HPA conversion in this strain. Indeed LFB1002 produced 3 mM less 1,3-PDO compared to the wild type, but the lower production did not correlate with 12 mM higher ethanol production, indicating a redox imbalance. However, samples from early stationary phase were analysed and the activity of the lr_1734 gene product in the late exponential growth phase is probably responsible for the additional 1,3-PDO production. Conversion of 3-HPA by cell-free extracts was similar for the wild type and LFB1001, but clearly lower in LFB1002, showing that 3-HPA conversion is indeed impaired in the latter and that the gene product of lr_0030 plays a major role in the conversion of 3-HPA to 1,3-PDO during exponential growth on glucose in the presence of glycerol. As the phenotypic differences between the wild type and LFB1002 were only observed in the presence and not in the absence of glycerol, our results strongly suggest that lr_0030 is also induced in MRS+glycerol, confirming data obtained in chemically defined medium (Table 3). In contrast, the role of lr_0030 during 3-HPA production seems to be minor, despite the presence of glycerol. The regeneration of NADH via 3-HPA to 1,3-PDO conversion provides only a benefit in the presence of glucose and as glucose is absent during 3-HPA production by resting cells, lr_0030 is probably down regulated.

ORF lr_1734 is situated in the glycerol utilization *pdu *operon. The organization of the *pdu *operon in *L. reuteri *is highly similar to that in *Salmonella *species [16], and therefore the lr_1734 gene product is likely involved in conversion of 3-HPA to 1,3-PDO. However, deletion of this gene had only limited impact on growth performance of *L. reuteri *in the presence of glycerol, whereas no altered end-product formation was observed. This is a puzzling observation, since lr_1734 is presumably transcriptionally coupled to the glycerol dehydratase genes (encoded by *pduCDE*). However, lr_1734 is located approximately 6 kB downstream of the *pduE *gene and polar effects could have a negative impact on the mRNA abundance of lr_1734. Consequently, limited cellular amounts of the encoded enzyme cannot cope with the abundance of glycerol dehydratase, resulting in limited NAD^+ ^regeneration and probably accumulation of toxic 3-HPA in the cell. Continuation of the metabolic flux therefore necessitates the activity of the second enzyme encoded by lr_0030 is necessary.

Alternatively, the product of lr_0030 could have a preference for NADP. During exponential growth the phosphoketolase pathway produces NADPH and co-factor regeneration could be more efficient if an NADP preferring 1,3-propanediol dehydrogenase is expressed. The enzymatic activities revealed clearly lower NADH conversion in the lr_0030 mutant compared to the wild type, showing that Lr_0030 can use NADH as substrate. However, NADPH oxidation by Lr_0030 is likely and the exact mechanism of regeneration of oxidizing equivalents during growth of *L. reuteri *in the presence of glycerol remains to be elucidated. Indeed LFB1002 produced 3 mM less 1,3-PDO compared to the wild type, although a high increase in ethanol production of 12 mM was recorded. This data suggests a redox imbalance. Samples from early stationary phase were analysed and data suggest that the activity of the lr_1734 gene product in the late exponential growth phase is likely responsible for additional 1,3-PDO production.

Lr_0030 is almost 3 times higher expressed in the presence of glycerol compared to growth without glycerol, whereas the effect of glycerol addition on lr_1734 expression was less notable (Table 3). The higher expression of lr_0030 correlates with the predominant role of the protein in 3-HPA to 1,3-PDO conversion during growth. The identification of this second 1,3-propanediol dehydrogenase provides new insight in glycerol conversion by *L. reuteri*.

Apart from its role as a substrate for regeneration of NAD^+^, 3-HPA is an anti-microbial compound assumed to play an important role in the ecology of *L. reuteri*. Although the mechanism of 3-HPA toxicity is partly unravelled [35,36], little is known about the self-defence mechanisms of the producer strains. Conversion of 3-HPA to 1,3-PDO might be a detoxification reaction for the cells. However, no differences in 3-HPA sensitivity was observed between the wild type and the two mutants (data not shown), suggesting that the function of the two enzymes encoded by lr_1734 and lr_0030 is restricted to 1,3-PDO conversion for NAD^+^.

## Conclusions

Our data provide new insight in the genes and enzymes involved in the conversion of 3-HPA to 1,3-PDO during growth on glucose plus glycerol in *L. reuteri*. We identified a gene product so far not associated with 3-HPA conversion and showed that the corresponding enzyme plays a pivotal role in 3-HPA conversion in exponential growing *L. reuteri *cells. Another gene product was shown to be involved in 3-HPA conversion in non-growing cells in a biotechnological production. The activities of this enzyme might be useful for future metabolic engineering processes to enhance production of both 3-HPA and 1,3-PDO.

Our study suggests that expression of paralogous genes enables fine-tuning of the metabolic flux in the cell and that different gene products are active under different circumstances as e.g. different growth conditions. These findings imply that the selection of targets in metabolic engineering strategies should be based on not only the primary biological function as annotated in the genome, but also on activity patterns of the corresponding enzyme.

## Authors' contributions

MS constructed the mutant strains, performed 3-HPA productions and enzymatic analyses, and drafted the manuscript. SV performed 3-HPA productions and purification, designed and supervised the study. LM participated in the design and supervision the study. CL coordinated the work, supervised and designed the study, and revised the manuscript. All authors have read and approved the final manuscript.

## Competing interests

The authors declare that they have no competing interests.

## Supplementary Material

Additional file 1 **Suppl. Figure 1: Construction of mutant strains LFB1001, LFB1002, and LFB1003**. A) Schematic overview of the double cross over event leading to replacement of the target gene by P_32_*cat*, exemplified for the ORF lr_1734. The approx. 1 kB up- and downstream regions of lr_1734 were cloned in the SwaI and Ecl136II site of pNZ5319 resulting in the gene replacement vector pLFB1002. The primers used for amplification are indicated by arrows in the wild type situation. Correct gene replacement was checked by PCR using primers annealing in the *cat *gene and outside the cloned regions. Control primers are indicated by arrows in the LFB1001 situation B) Gel electrophorese showing the PCR products that confirm correct genetic reorganization: Lane 1: control lr_0030 upstream; Lane 2: control lr_0030 downstream; Lane 3: 100 bp marker (Bioconcept, Allschwil, Switzerland); Lane 4: 1 kB marker (Bioconcept); Lane 5: control lr_1734 upstream; Lane 6: control lr_1734 downstream. Click here for file
