# Supplementary material for: 1,3-Propanediol dehydrogenases in Lactobacillus reuteri: impact on central metabolism and 3-hydroxypropionaldehyde production
Source: Microb Cell Fact. 2011 Aug 3;10:61. doi: 10.1186/1475-2859-10-61 (PMC3180264; doi:10.1186/1475-2859-10-61)
Supplement: Additional file 1 — Suppl. Figure 1: Construction of mutant strains LFB1001, LFB1002, and LFB1003. A) Schematic overview of the double cross over event leading to replacement of the target gene by P32cat, exemplified for the ORF lr_1734. The approx. 1 kB up- and downstream regions of lr_1734 were cloned in the SwaI and Ecl136II site of pNZ5319 resulting in the gene replacement vector pLFB1002. The primers used for amplification are indicated by arrows in the wild type situation. Correct gene replacement was checked by PCR using primers annealing in the cat gene and outside the cloned regions. Control primers are indicated by arrows in the LFB1001 situation B) Gel electrophorese showing the PCR products that confirm correct genetic reorganization: Lane 1: control lr_0030 upstream; Lane 2: control lr_0030 downstream; Lane 3: 100 bp marker (Bioconcept, Allschwil, Switzerland); Lane 4: 1 kB marker (Bioconcept); Lane 5: control lr_1734 upstream; Lane 6: control lr_1734 downstream. [file 1475-2859-10-61-S1.DOC]

**Suppl. Fig. 1: Construction of mutant strains LFB1001, LFB1002, and LFB1003.** A) Schematic overview of the double cross over event leading to replacement of the target gene by P_32_*cat*, exemplified for the ORF lr_1734. The approx. 1 kB up- and downstream regions of lr_1734 were cloned in the SwaI and Ecl136II site of pNZ5319 resulting in the gene replacement vector pLFB1002. The primers used for amplification are indicated by arrows in the wild type situation. Correct gene replacement was checked by PCR using primers annealing in the *cat* gene and outside the cloned regions. Control primers are indicated by arrows in the LFB1001 situation B) Gel electrophorese showing the PCR products that confirm correct genetic reorganization: Lane 1: control lr_0030 upstream; Lane 2: control lr_0030 downstream; Lane 3: 100 bp marker (Bioconcept, Allschwil, Switzerland); Lane 4: 1 kB marker (Bioconcept); Lane 5: control lr_1734 upstream; Lane 6: control lr_1734 downstream

**A**

**B**
